# Design and Implementation of a Community-Based Educational Program to Enhance Prostate Cancer Screening in Southeastern Puerto Rico

**DOI:** 10.3390/healthcare13212749

**Published:** 2025-10-30

**Authors:** Juan Derieux-Cruz, Milton Rodríguez-Padilla, Yaritza Pérez, Luis Arroyo-Andújar, Gilberto Ruiz-Deyá, Jaime Matta, Melissa Marzán-Rodríguez, Julio Jiménez-Chávez

**Affiliations:** 1 School of Behavioral and Brain Sciences, Ponce Health Sciences University, Ponce 00716-2347, Puerto Rico; jderieux22@stu.psm.edu (J.D.-C.); yaperez20@stu.psm.edu (Y.P.); 2 Graduate School of Public Health, Medical Sciences Campus, University of Puerto Rico, San Juan 00936-5067, Puerto Rico; milton.rodriguez1@upr.edu (M.R.-P.); luis.arroyo@upr.edu (L.A.-A.); 3 Department of Surgery, Ponce Health Sciences University, Ponce 00716-2347, Puerto Rico; gruiz@psm.edu; 4 Department of Basic Sciences, Divisions of Pharmacology and Toxicology, Cancer Biology, Ponce Research Institute, Ponce Health Sciences University, Ponce 00716-2347, Puerto Rico; jmatta@psm.edu; 5 Public Health Program, Ponce Health Sciences University, Ponce 00716-2347, Puerto Rico; mmarzan@psm.edu; 6 Specialized Center in Health Disparities, Ponce Research Institute, Ponce Health Sciences University, Ponce 00716-2347, Puerto Rico

**Keywords:** prostate cancer early detection, health literacy, community-based educational intervention, Intervention Mapping, prostate cancer screening uptake, self-care practices

## Abstract

Background/Objectives: Prostate cancer (PCa) has the highest incidence and mortality rates among men in Puerto Rico. However, screening and early detection programs remain limited and fragmented. This study presents the design and implementation of a community-based educational program to increase PCa screening and knowledge in three southeastern rural communities with high African ancestry and elevated PCa mortality. Methods: Conducted between 2021 and 2025, this mixed-method study followed Community Engagement principles and was guided by the Intervention Mapping framework. A Community Advisory Committee informed each step of the intervention, which included PSA and digital rectal examination (DRE) testing via a mobile clinic staffed by urologists. Pre- and post-tests measured knowledge gains and willingness to screen, while satisfaction surveys evaluated the program’s impact. Results: After the intervention, knowledge scores increased significantly (t = −5.5, *p* < 0.001), and 76% of participants reported greater confidence in making health decisions. In total, 95 men accessed screening services through a mobile clinic, 33 were referred for follow-up, and 4 PCa cases were detected. Conclusions: Combining culturally tailored education with accessible screening helped overcome sociocultural and structural barriers, showing promise for reducing PCa disparities in underserved Puerto Rican populations.

## 1. Introduction

Prostate cancer (PCa) is the second most diagnosed cancer and the fifth leading cause of cancer-related death among men worldwide, with approximately 1.4 million new cases and 97,000 deaths reported in 2023 [1]. Risk factors include age, diet, obesity, metabolic syndrome, ancestry, family history of PCa, and specific genetic mutations [2]. In Puerto Rico (PR), a U.S. territory in the Caribbean with an estimated population of 3.2 million, PCa is the cancer with the highest incidence and mortality among men [3,4], with about 17% expected to develop the disease during their lifetime [5]. Compared to men in the continental United States, Puerto Rican men are disproportionately affected by PCa [6], a disparity associated with genetics, African ancestry, socioeconomic disadvantage, limited healthcare access, lifestyle, and environmental exposures. These challenges occur within a broader context of poverty, inadequate infrastructure, a weakened healthcare system, and regional environmental risks [7]. Despite this burden, educational and screening programs for PCa are scarce or absent across the 78 municipalities. Unlike breast cancer, which benefits from consistent awareness campaigns, Puerto Rico lacks coordinated, evidence-based educational initiatives to promote early detection and reduce PCa disparities.

Countries with higher income levels tend to exhibit the highest cancer incidence rates, yet they also demonstrate the most significant reductions in cancer-related mortality [8]. In contrast, lower-income countries generally report lower incidence but face disproportionately higher mortality rates, which highlights disparities in diagnostic practices and healthcare infrastructure.

PCa can be divided into early-stage and advanced-stage disease, including metastasis to other organs. Indolent PCa tumors grow slowly and may not cause significant adverse effects over a patient’s lifetime [2]. In contrast, advanced PCa often progresses rapidly (2–3 years) despite androgen deprivation therapy and can become castration-resistant (mCRPCa) and lethal [9]. Early PCa detection is associated with a more favorable prognosis and longer 5- and 10-year survival rates. This underscores the necessity for public education and early detection efforts, particularly among individuals over 40, to identify early-stage PCa when treatments are more effective, less invasive and costly. Additionally, genetic predisposition plays a significant role in the aggressiveness of PCa. The rs72725854 genetic variant, which is highly prevalent in men of African descent, significantly increases the likelihood of developing aggressive and lethal PCa [10]. This finding highlights the need for personalized detection strategies tailored to high-risk communities. 

Early detection of PCa plays a crucial role in reducing mortality factors and improving patients’ quality of life. Therefore, the primary goal of early detection is to identify PCa at a stage when it requires less invasive treatment with fewer adverse effects and a higher probability of a cure [11]. Moreover, studies such as the European Randomized Study of Screening for PCa (ERSPC) have provided level 1 evidence demonstrating that prostate-specific antigen (PSA) screening reduces PCa-specific mortality by 20% after 16 years of follow-up and metastatic disease by 30% after 12 years [12]. Combining PSA with risk calculators enables more accurate risk stratification, thereby reducing unnecessary biopsies and overdiagnosis [12,13]. In this regard, educational programs focused on early detection have proven effective in increasing knowledge, perceptions, and intent to screen, directly improving health outcomes [14,15,16]. For instance, educational interventions targeting PCa have significantly increased screening rates and supported informed decision-making [17]. 

According to the American Cancer Society [3], the prevalence of PSA screening in PR in 2021 was 48%, which surpassed the average of 44% in the United States among men who had not been diagnosed with PCa. Nevertheless, there is a paucity of programs designed to facilitate the early detection of PCa, and those that do exist lack sufficient centralization [18,19,20]. This study aims to develop and implement a community engagement educational program to enhance knowledge and screening for PCa in three southeastern Puerto Rican communities: Maunabo, Patillas, and Las Piedras. These communities were selected based on three key factors: their elevated PCa mortality rates, elevated African ancestry [21,22], and low socioeconomic status. 

## 2. Materials and Methods

**Figure 1:**
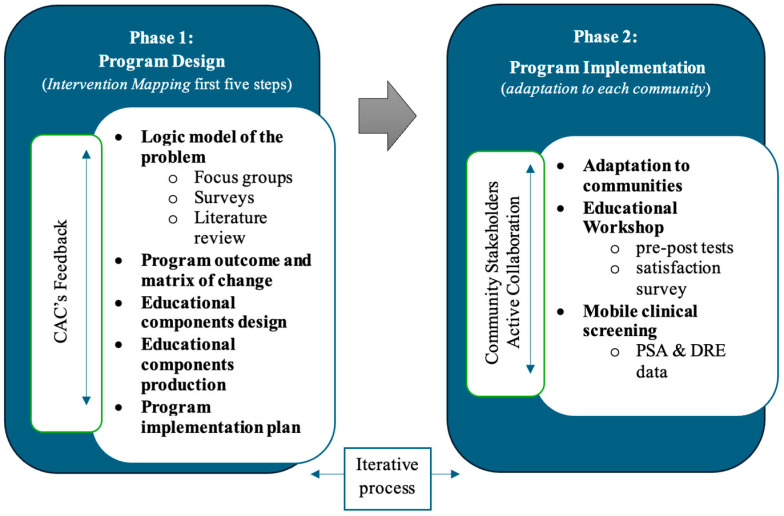
Study phases: Design and Implementation.

The present study focused on three municipalities in the southeastern region of PR: Patillas, Maunabo, and Las Piedras. These communities were selected based on their high PCa mortality rates, elevated African ancestry proportions, and persistent socioeconomic challenges. Patillas and Maunabo are coastal, predominantly rural towns characterized by limited healthcare infrastructure and a high prevalence of transportation difficulties. Las Piedras, while somewhat more urbanized, remains a rural municipality that also faces significant health disparities. This study employed a quasi-experimental pre-post design to evaluate the feasibility and preliminary impact of a community-based educational program to increase PCa knowledge and screening behaviors (references provided for these methods). This study was conducted between 2021 and 2025 and consisted of two phases: intervention design and program implementation, as summarized in Figure 1.

The first phase involved developing the intervention by following the first five steps of the Intervention Mapping (IM) framework [23]. Researchers conducted focus groups and community surveys to identify knowledge gaps, cultural beliefs, and screening obstacles.

A Community Advisory Committee (CAC) of seven men from the target municipalities guided all stages of the process.

- CAC Composition:
  ○ Two PCa survivors.
  ○ Two family members/caregivers.
  ○ Two representatives from community or faith-based organizations.
  ○ One healthcare provider.
- CAC Main roles:
  ○ Provide ongoing feedback to ensure cultural and contextual relevance.
  ○ Identify key priority areas (e.g., limited education, “machismo,” access barriers).
  ○ Support recruitment by engaging community leaders and selecting meeting sites.
  ○ Review preliminary findings and refine educational materials and workshop content.
  ○ Assist in logistics and recruitment during implementation.

Several meetings were held with the CAC throughout the intervention planning and development phases. In the early stages, the CAC helped identify key priority areas—such as lack of education, “machismo,” and limited access to healthcare—to guide the content of focus groups and surveys. Machismo has been defined as a cultural norm that places immense pressure on men to maintain a strong and stoic image, which can hinder open communication about prostate cancer experiences [24].

The CAC also supported the planning of the focus groups by identifying community leaders to assist with recruitment and by recommending appropriate locations, dates, and times to maximize participation. Afterwards, the CAC provided feedback on preliminary findings and assisted with the logistics of survey administration in the community. Seven focus groups (*n* = 63 men) were held between December 2021 and April 2022 in Maunabo (2), Patillas (2), and Las Piedras (3), each with 10 or more participants. Although Krueger and Casey [25] recommend an ideal size of five to eight participants to balance participation and diversity of perspectives, groups of around ten were planned to account for possible absences on the day of the session and to ensure sufficient representation from all community sectors. In addition, at least two focus groups were conducted per municipality to facilitate data triangulation and capture the range of perspectives within each community context.

Community leaders identified potential participants, who were then contacted by the research team. Before beginning the sessions, informed consent was explained and signed by all participants. The discussions, conducted by trained facilitators, lasted between 60 and 90 min. Data saturation was achieved progressively throughout the process, as common patterns and no new themes emerged by the final focus groups conducted in Las Piedras. Lastly, members reviewed a draft of the educational components (workshop and educational materials) program, offering suggestions to improve its relevance and delivery, and supported recruitment efforts for the intervention’s implementation phase.

The second phase consisted of the intervention’s implementation in the three selected low-income communities (Patillas, Maunabo, and Las Piedras). This phase included educational workshops and clinical screenings delivered in partnership with community stakeholders. Participants completed pre-and post-tests to assess knowledge gains, along with a satisfaction survey containing Likert-scale and open-ended items. Simultaneously, a mobile clinic provided PCa screenings, where the team recorded the number of attendees evaluated by urologists and nurses, the number of PSA and DREs conducted, and the identification of suspected tumor cases.

This study targeted men aged 40 and older from Patillas, Maunabo, and Las Piedras. Participants were recruited through community organizations, local leaders, and health centers. Prior to their participation in this study, all participants in Phase 1 provided informed consent. This study was reviewed and approved by the Institutional Review Board (IRB) of the Ponce Research Institute (Protocol #21015245, 18 February 2021), ensuring adherence to ethical standards for research involving human subjects.

### 2.1. Phase 1: Development of the Intervention (Intervention Mapping)

#### 2.1.1. Step 1: Logic Model of the Problem

The CAC identified community needs and provided insights into men’s habits, perspectives on PCa, attitudes, and limitations to early detection practices. Additionally, the CAC collaborated closely with the research team to develop, coordinate, and implement the qualitative and quantitative phases of the needs assessment. The CAC’s insights on local beliefs, common obstacles, and PCa risks shaped the educational content and intervention design, ensuring they were culturally relevant and appropriate for the target communities.

Building data from the CAC, the team and community members co-developed a mixed-method study. This study used focus groups (*n* = 63) and surveys (*n* = 144) to assess knowledge about PCa, cultural beliefs, perceived risks, and obstacles to early detection. The results of this needs assessment shaped the program by identifying specific gaps and misconceptions in the community’s understanding of PCa. Researchers conducted a literature review alongside this process, revealing the critical role of culturally tailored interventions in improving screening uptake, the importance of addressing structural limitations, and the value of community-driven educational efforts. This research highlighted that effective PCa screening programs must integrate both evidence-based strategies and local cultural contexts to achieve optimal outcomes.

#### 2.1.2. Step 2: Program Outcome and Matrix of Change

From these findings, the research team determined the program’s primary objectives, focusing on addressing the identified obstacles. These objectives included reducing resistance to screening by informing participants about PCa, associated risk factors, and the significance of early detection. The team offered a free community-based educational workshop. Additionally, the program aimed to provide free PCa screenings through the Ponce Health Sciences University/Ponce Research Institute (PHSU/PRI) clinic, which operated immediately after the workshop. The mobile clinic gave participants direct access to urology specialists and nurses, addressing the limitation to specialized care access. Nurses obtained blood samples for PSA screening.

#### 2.1.3. Step 3: Design of the Educational Components

After analyzing the assessment data with the CAC, the research team identified several key topics for inclusion in the workshop. They selected topics such as statistics related to PCa, fundamental concepts, available screening tests, common obstacles to screening, and men’s self-care. The team designed the community engagement program to address the specific needs identified in the community assessment, including education and access to specialized care services.

For the educational component, the team adapted the Cancer 101 educational program. Cancer 101 is an NCI-funded cancer education initiative developed initially for American Indian and Alaska Native communities [26,27]. Research has demonstrated that this program increases cancer awareness, fosters positive attitudes toward preventive behaviors, and promotes proactive health management in diverse populations, including Puerto Ricans [27,28]. The adapted version for the Puerto Rican population was the foundation for the workshop’s structure, thematic sequence, and content.

The Theory of Planned Behavior (TPB) also guided the workshop’s content, predicting how individuals respond to health challenges. TPB explains that behavior is driven by intentions, which are shaped by attitudes, subjective norms, and perceived control. The team integrated these three focal points into the workshop to address obstacles that hinder early detection of PCa. They designed the content to assess participants’ perceptions of screening benefits, the influence of social norms, and the perceived ease or difficulty of undergoing screening. Finally, the workshop delivered essential information on PCa, including epidemiological data, symptoms, risk factors, diagnostic tests, the benefits of early detection versus late diagnosis, and self-care concepts.

The psychoeducational workshop consisted of a single 40 min session delivered as one structured module divided into five thematic segments: (1) cancer in Puerto Rico—statistical overview; (2) prostate cancer—key concepts and risk factors; (3) screening and detection tests; (4) barriers to early detection and cultural beliefs; and (5) a short trivia activity to reinforce learning. The content was tailored to the Puerto Rican context, previously evaluated by the CAC. The workshop was taught in colloquial Puerto Rican Spanish and designed with adapted health education strategies, integrating accessible colloquial language and creating a participatory environment. The session was conducted entirely in Puerto Rican Spanish to ensure linguistic and cultural relevance. Three trained facilitators—a psychiatrist, a health educator and a psychology doctoral trainee—led the workshop using PowerPoint slides, printed educational materials, and interactive discussions. The workshop emphasized culturally resonant examples and closed with a group reflection on the importance of early detection and proactive health decisions.

#### 2.1.4. Step 4: Educational Components Production

The research team refined the program structure and materials based on input from the CAC and expert consultation with a urologist who was part of the study team. They developed educational materials in brochures and booklets to increase awareness of PCa, the screening process, and self-care behaviors. All materials and the workshop were developed and delivered in Spanish to ensure cultural and linguistic appropriateness. Additionally, the team provided many medical evaluations, clinical examination results (including PSA), and upcoming medical appointments to empower participants to track their health screenings over time.

Building on this behavioral framework, the team incorporated the Health Belief Model (HBM) to enhance the workshop’s impact. The HBM provides an additional perspective for understanding and influencing PCa screening behaviors. Educational interventions based on HBM have shown promising effectiveness, increased awareness, and promoted proactive health decisions.

To ensure alignment between theoretical and practical considerations, the team designed brochures and booklets to reflect the workshop’s objectives. These materials aimed to facilitate knowledge acquisition, increase awareness of PCa screening, and address the misconceptions identified within the focus groups. The principal topics covered included: the definition of lethal PCa, the purpose, timing, and methods of screening, the differences between early and late detection, risk factors, common symptoms, relevant local statistics, and self-care recommendations. Additionally, attendees received a list of recommended questions to ask their physician in the event of a diagnosis, enabling them to take an active role in their own health care.

The materials also included a practical tool: a table for participants to record upcoming screenings, test dates, results, and clinician recommendations. To accommodate different uses, the team produced the materials in two formats: a brochure and a booklet. The brochure allowed for easy distribution to family members and others, while the booklet served as a self-care diary, including an expanded six-page logbook to track follow-ups over several years.

#### 2.1.5. Step 5: Program Implementation Plan

To coordinate the program, the team required extensive communication with supporting organizations and resources. They conducted numerous calls and meetings to ensure seamless implementation. For example, their collaboration with one of the healthcare centers played a key role in organizing the logistics of the mobile clinic. Additionally, meetings with the CAC and local leaders helped identify optimal locations and promotional strategies, ensuring active involvement from community-based organizations.

To refine the program further, the team held several meetings with the CAC to share ideas and gather input. These meetings were crucial for identifying contacts within municipal governments for potential support and collaboration. The research team, joining CAC members, explored possible locations and more convenient schedules for workshops, considering the need to conduct activities in both urban and rural areas. They discussed promotional strategies, such as using local radio stations and newspapers to expand the activity’s outreach.

Building on these discussions, the team organized three complementary meetings in each municipality to finalize promotional activities. In the first meeting, a community leader facilitated communication with Maunabo’s radio station to promote the event. In the second meeting, another community leader agreed to distribute promotional materials through WhatsApp community groups and provided contact information for a radio program affiliated with a health center in Patillas, where the intervention was later conducted.

### 2.2. Phase 2: Implementation of the Program

The team implemented the program following a structured process to ensure its feasibility and alignment with community needs. With assistance from CAC members, they identified key stakeholders, including community leaders, healthcare providers, and municipal representatives. These individuals played a crucial role in recruiting participants, selecting accessible and convenient locations for the intervention, and promoting the program through radio programs, newspapers, and social media. Additionally, the team determined the most suitable days and times to optimize community participation and coordinated visits to intervention sites to ensure seamless implementation.

The team successfully implemented the program in Patillas, Maunabo, and Las Piedras. To assess its impact, they administered a series of pre-and post-tests to participants, evaluating changes in knowledge and behavior. The pre- and post-test consisted of 12 closed-format items, with three answer alternatives (“true”, “false”, and “I don’t know”), and a score ranged from 0 to 12. These items were specifically prepared for the intervention and submitted for review by the Community Advisory Committee. The aim was to guarantee their cultural relevance, linguistic clarity, and alignment with the workshop’s contents, providing evidence of content validity. We recognize that, as it is an instrument developed for this study, its formal psychometric properties are limited; however, its internal consistency was adequate in a community context. The results have also been strengthened by including 95% confidence intervals and effect size, to communicate not only statistical significance, but also the magnitude of the observed change. Additionally, they distributed a brief satisfaction questionnaire, gathering feedback to help refine the program and ensure it continued meeting community needs.

Implementation strategies were adapted to the unique contexts of each municipality. In Maunabo, activities were conducted in the municipal legislature hall in the town center and in a rural mountain community, allowing for broader geographic reach. In contrast, the Patillas and Las Piedras interventions were held in local health centers. In these two municipalities, additional strategies were employed to facilitate participation from individuals without access to transportation, including support from community leaders and coordination of local transport options.

To guide the program’s design and evaluation, the team carefully selected assessment instruments, including socio-demographic surveys and structured interviews with 10 individuals (5 administrators and 5 community members). They planned to conduct these interviews in subsequent phases to collect valuable insights that would help keep the intervention relevant, responsive, and aligned with community needs.

A mixed-method approach was employed to analyze the data. All audio recordings were transcribed and analyzed qualitatively using NVivo, a qualitative data analysis software. Quantitative data analyses were conducted using IBM SPSS Statistics version 29 (IBM Corp., Armonk, NY, USA). Descriptive and inferential statistics were applied, with the level of statistical significance set at *p* < 0.05.

## 3. Results

The findings of this study are presented via two principal phases, reflecting the distinct stages of the intervention mapping process. The first phase presents the needs assessment results, which were conducted as the preliminary step to identifying obstacles, community perceptions, and preferences related to PCa screening. Based on focus groups and surveys, this assessment provided a comprehensive understanding of the sociocultural and practical challenges the target communities face. The second phase presents the outcomes of the implemented interventions, including educational programs and clinical screenings, which were designed to address the identified needs and limitations. These results demonstrate the impact of tailored community engagement strategies on increasing knowledge, improving attitudes, and facilitating access to PCa screening and follow-up care. These findings offer valuable insights into the preliminary impact of a structured approach to address health disparities in underserved communities.

### 3.1. Phase 1: Development of the Intervention Program

The needs assessment for the PCa screening program was conducted by using focus groups during December 2021 to April 2022. A total of 63 men participated in the focus groups conducted in the municipalities of Patillas, Maunabo, and Las Piedras. All 63 participants were residents of said municipalities, with ages ranging from 41 to 90 years (52% of participants reported to be 65 or older, *n* = 33). Regarding civil or relationship status, participants reported being “Married” (54%, *n* = 34), “Single” (16%, *n* = 10), “Living together but not legally married” (13%, *n* = 8), “Divorced” (11%, *n* = 7), and “Widowed” (6%, *n* = 4). Concerning educational level, 37% (*n* = 23) of participants reported to have completed high school or lower, including eight participants (34.78%, *n* = 8/23) who reported having only reached some elementary grades. The other 63% (*n* = 40) of participants reported having a degree after high school (associate degree, *n* = 3; bachelor’s degree (*n* = 18), master’s degree (*n* = 3), doctorate degree (*n* = 5)). Finally, 41% of participants reported having a monthly household income of less than $1500.

Difficulties in PCa screening were identified during the focus groups. Key obstacles included cultural perceptions such as “machismo,” which fosters resistance to screening by framing prostate exams as invasive in intimate areas considered “secret and private.” This cultural perception contributes to fears of losing virility and enduring social judgment, such as ridicule, criticism, or negative comments from others. Additional difficulties included limited access to healthcare services, lack of health insurance, and insufficient availability of medical specialists. Psychological obstacles were also prevalent, such as fear of being diagnosed with cancer and concerns about experiencing pain during the screening process. Specifically, four themes associated with “machismo” emerged as significant cultural obstacles to PCa screening, as detailed in Table 1.

These findings illustrate how cultural and psychological obstacles, rooted in the concept of machismo, intersect to discourage men from undergoing potentially lifesaving PCa screening. By addressing these issues and integrating culturally sensitive interventions, healthcare initiatives can better overcome these obstacles and promote PCa screening in under-supported communities.

The distribution of participants included 34% from Patillas (*n* = 49), 33% from Maunabo (*n* = 47), and 33% from Las Piedras (*n* = 48). A summary of their socio-demographic characteristics and screening attitudes is presented in Table 2. The data provides an overview of participants’ health insurance coverage, marital status, prostate-related health history, income levels, educational attainment, and perceptions regarding the usefulness of PSA screening. This information offers important context for understanding the characteristics of the community involved in this study.

The Masculinity in Chronic Disease Inventory (MCD-I) [29,30,31], (completed by 120 participants, showed a high level of “machismo” among respondents (99%). The reliability of the MCD-I was examined using Cronbach’s alpha, the standardized Cronbach’s alpha, and the alpha coefficient after deleting items. The Cronbach’s alpha for the full 19-item scale was 0.87, and the standardized alpha was 0.88, indicating strong internal consistency. All items were retained in the final version since none negatively affected the overall reliability of the scale.

Additionally, the results of a Prostate Cancer Knowledge Questionnaire developed by the research team showed that 97% of respondents had a low level of knowledge about prostate cancer. The questionnaire had a Cronbach’s alpha of 0.69, which improved to 0.71 when it was reduced from 12 to 11 items.

Regarding receiving health information, 66% (*n* = 95) of participants preferred to receive it through printed educational material (61%, *n* = 89), educational talks (52%, *n* = 75), radio or television advertisements (44%, *n* = 30), and newspaper advertisements (23%, *n* = 33).

### 3.2. Phase 2: Implementation

The sociodemographic profile of the educational intervention consisted of 75 participants from municipalities: Maunabo (*n* = 20, 27%), Patillas (*n* = 40, 53%), and Las Piedras (*n* = 15, 20%). Most of the participants were 65 years of age or older (*n* = 31, 55%) and had monthly incomes ranging from $501 to $1000 (*n* = 18, 31%) or $1001 to $1500 (*n* = 16, 28%) (Table 3). Most participants were married (*n* = 29, 50%) and had a high school diploma as their highest level of education (*n* = 26, 46%).

**Figure 2:**
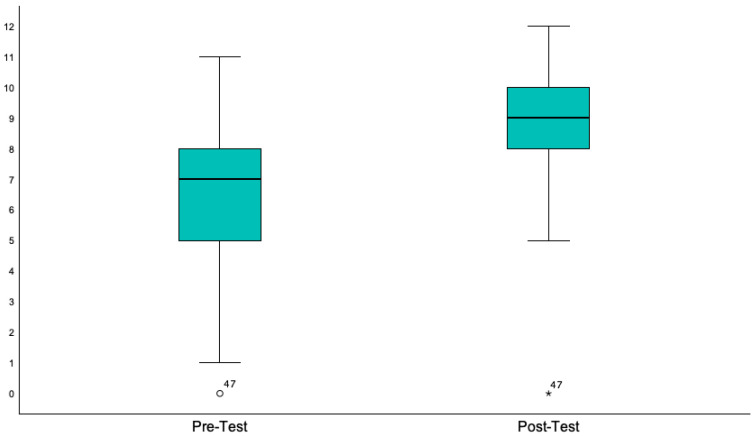
Comparison of participants’ knowledge scores before and after the educational workshop. A paired samples *t*-test demonstrated a statistically significant improvement (*p* < 0.001), with median scores increasing and score variability decreasing post-intervention. Participant 47 was identified as an outlier in both the pre-test (circle) and the post-test (asterisk), with a score of 0 in each case.

The Educational Community Engagement Program demonstrated feasibility and promising results by significantly improving participants’ knowledge scores from pre-test to post-test. Assessing the impact of the intervention, we observed a significant improvement in PCa knowledge and screening practices, with pre-test results (M = 6.8, SD = 2.4) and post-test results (M = 8.5, SD = 2.2) showing a notable increase, t(59) = −5.5, *p* < 0.001, 95% CI [−1.18, −0.60], d = −0.89. As illustrated in Figure 2, post-test scores increased significantly following the educational intervention.

Participants’ evaluations of the educational program were very positive. Overall, 84% of respondents liked the way the information was presented, while 80% agreed that they learned something new, and 76% felt able to make healthier choices after attending. Additionally, 81% enjoyed the activity, 74% felt motivated to share what they learned with others, 77% were willing to return for similar activities, and 77% agreed that the program was well-organized, and the duration was appropriate. Qualitative feedback further supported these findings, highlighting the clarity and well-structured nature of the explanations, the adequacy and simplicity of the educational materials, the professionalism and strong performance of the speaker, and the relevance and well-presented nature of the information. Participants also provided suggestions for improvement, such as offering more sessions throughout the year, adjusting the schedule to allow for greater participation, expanding discussions with additional talks and clarifications, and starting sessions earlier. This feedback highlights the program’s promising impact while offering valuable insights for future enhancements.

The mobile unit clinical intervention involved 95 participants who were screened by urologists and nurses during the previous interventions in the targeted communities. Of these participants, 67 received a PSA test, 53 received a DRE, and four were identified with suspicious positive findings. Participants could receive more than one type of test based on their individual needs and physician recommendations.

In addition, 33 men were referred for follow-up care. Immediate appointments with local urologists and transportation were arranged for those who lacked the resources to attend their scheduled follow-up appointments. This comprehensive approach ensured participants had access to necessary diagnostic and clinical care services.

## 4. Discussion

In Puerto Rico PCa is the cancer with the highest incidence and mortality in men according to the Puerto Rico Cancer Registry (rcpr.org) [3]. However, there is greater visibility, promotion of self-care, and attention to other types of cancer such as breast cancer. This difference highlights the need to focus efforts on raising awareness and improving attitudes towards early screening for PCa.

Emerging research and changes in medical guidelines coupled with the positive impact of educational interventions have generated updated protocols on PCa screening. However, there is an important need for dissemination to the community [14,15,16]. For example, the European Randomized Study of Screening for Prostate Cancer (ERSPC) demonstrated a 21% reduction in PCa mortality after 13 years of follow-up due to screening interventions [32]. Addressing this need, this study focused on developing an intervention program to increase knowledge and improve attitudes toward PCa screening in men from underserved communities in the southeastern region of Puerto Rico.

The MI approach was used to ensure that the program’s design, implementation, and evaluation systematically and iteratively addressed multiple factors that can influence beliefs and behaviors associated with PCa. IM has been used successfully in other contexts, including community-based cancer prevention programs [19,33,34]. One of IM’s strengths is its emphasis on keeping the community’s voice active throughout the intervention development process. Community participation is key to achieving culturally sensitive educational programs with a greater chance of being better accepted during implementation [35]. However, the literature [36] indicates that less than half of community studies explicitly document input from community members, reinforcing our interest in maintaining their participation. This article describes the first five steps of IM; the sixth stage, corresponding to the follow-up evaluation, is currently ongoing.

The intervention was carefully tailored to the sociocultural context to comprehensively address the identified obstacles by integrating the opinions, ideas, and concepts of community members, healthcare providers, cancer survivors, and other key stakeholders. We used a mixed-method approach, combining focus groups and surveys to collect community input.

From the beginning of the process, the CAC’s participation was instrumental, a strategy widely recognized in the literature [37,38,39,40,41,42,43,44]. Our CAC, comprising seven people with diverse characteristics (PCa survivors, caregivers, community organization leaders, and a health care provider), reflected multiple perspectives related to PCa. The CAC contributed significantly to developing question guides, identifying obstacles, recruiting participants, promoting this study, and connecting with local leaders. The CAC was often a facilitator in establishing alliances with key figures such as administrators and providers of local health centers, media (radio, newspapers), and community-based and faith-based organizations. These alliances were decisive when implementing the intervention program, favoring the acceptance and legitimacy of the intervention in the target communities.

Our first step was to evaluate the factors that act as obstacles to screening for PCa in men over 40 years of age from low-income communities. We identified “machismo,” fear of diagnosis, limited access to health services, and lack of education on preventive issues as the main obstacles. This information served as the central basis for designing the culturally sensitive educational program, which was adjusted to the characteristics of the male population of the target communities.

Undoubtedly, limited access to specialized services in rural areas is a recognized problem [45]. A major innovation in our study was the incorporation of a clinical component through the mobilization of a clinical unit and integration of urologists and nurses during community visits. This strategy helped reduce obstacles to access by directly offering clinical services in family environments. We observed that holding these activities in spaces recognized as safe and reliable by the community (primary health centers) and the direct provision of clinical services (evaluation by urologists) were key factors in responding to the program’s implementation. The results indicate that the interventions, both the psychoeducational workshop component (the knowledge gained can be added) and the clinical evaluation (clinical examination, PSA, and DRE), were very well accepted by men; perhaps because the program addressed structural and psychosocial obstacles in an integrated manner.

A highlight of our experience was the role of women in facilitating men’s positive response to participating in the program. Culturally, in our communities, women tend to motivate their partners, children, or family members to go to the doctor. This dynamic was evident in the activities, where women accompanied men and waited outside the clinics. Recognizing their role, some women also occasionally received educational information on the early detection of prostate cancer. In one of the communities, a community leader spontaneously proposed establishing a women’s committee to encourage healthy behaviors by men. From this experience, we suggest considering the role of women as a powerful strategy to promote male self-care. Although “machismo” persists as a cultural obstacle, female leadership can be a valuable bridge to encourage male participation in educational activities.

One important suggestion from workshop participants was to create a digital version of the psychoeducational intervention that can be shared through social media and online platforms. This adaptation could expand the reach, especially among younger audiences and people with transport limitations, thus complementing face-to-face efforts [46]. Digital tools can potentially increase awareness and participation, especially among younger people, while complementing the existing community approach [47].

In addition, we recommend developing specific guidelines to encourage male participation in health research and educational programs. Concrete strategies include engaging local male figures as speakers of educational messages, designing visual campaigns that reinforce a positive image of healthy masculinity, and organizing male-led community workshops.

A limitation of our study is that it was only conducted in three municipalities. In addition, our evaluation focused on short-term results (knowledge gained and satisfaction). Future research should assess long-term behavior change, screening rates, and the intervention’s sustained impact. Longitudinal studies will allow screening and self-care behaviors to be evaluated over time, providing insight into the long-term sustainability of the program’s impact.

Additionally, although the Masculinity in Chronic Disease Inventory (MCD-I) has shown high internal consistency in its original version (α = 0.87), its application in the present sample should be interpreted with caution, given that there are no psychometric validation studies available in the Puerto Rican population. This absence of evidence of cultural invariance represents a methodological limitation and an important area for future research.

Moreover, expanding the program to other regions of Puerto Rico will facilitate comparative analyses of obstacles and outcomes, allowing for tailored interventions to meet specific regional needs.

## 5. Conclusions

Our findings reinforce the importance of integrating community participation in the design of health interventions using culturally relevant strategies to address the challenges of cancer prevention and early detection. To strengthen the program’s sustainability, we suggest strategies such as training community health promoters, forming partnerships with municipalities, local health centers, and universities, and seeking local or community funding. With further evaluation and development, this program has the potential to contribute significantly to improving the early detection of prostate cancer in various settings.

## Figures and Tables

**Table 1:** Focus Group Themes Reflecting Masculinity-Related Resistance to Prostate Cancer Screening.

Theme	Description	Illustrative Quotes
Social judgment (fear of social criticism, threat to the social image of gender)	Men expressed concern over societal perceptions of masculinity and the judgment associated with undergoing digital rectal exams (DRE).	a. “…Machismo in our society and perhaps, in a great number of societies, machismo kills us.” b. “You don’t like it [DRE], you don’t want to be judged by other people.” c. “We live in a society that does not live life, and we are pending of what others say.”
Bodily invasion (fear of digital rectal exam [DRE], exposure of intimate areas related to masculinity/virility, fear of pain)	The physical nature of the DRE was perceived as a violation of personal and masculine boundaries.	a. “The machismo that we have in us. Sometimes, if the doctor does a rectal test, we don’t go.” b. “Most of us go because they do a blood test, but when it’s the rectal, people say, ‘No, I’m not going there.’”
DRE stigmatization (fear of being associated with marginalized groups)	Stigmas related to masculinity and gender identity also emerged as obstacles, with men fearing associations with marginalized groups or loss of their masculine identity.	a. “Most of us go because they do a blood test, but when it’s the rectal, people say, ‘No, I’m not going there.’” b. “Well, you know how the tests they give you put their finger in... Sometimes it’s about convincing the man (referring to the DRE) that you’re not going to lose an ounce of manhood.”
DRE-related jokes (a mechanism to hide the fear of diagnosis)	Humor and jokes served as coping mechanisms to mask deeper fears of diagnosis or the procedure itself.	a. “A lot of jokes have been created, a lot of hesitancy in relation to this type of exam.” b. “Because I had never been to a urologist, for the same joke situation, you know how that is. It is fear of facing the doctor to be told that you have this. Fear of diagnosis.”

**Table 2:** Socio-demographic Characteristics and Screening Attitudes of Survey Participants (*N* = 144).

Characteristic	Total (%)
Public health insurance	53%
Private health insurance	30%
Married	70%
Prostate-related problems (self-reported)	35%
Prior prostate cancer diagnosis	8%
Annual family income < $35,000	~55%
High school education or lower	68%
Perceive PSA test as useful for screening	59%

**Table 3:** Sociodemographic Profile of participants in the educational interventions in Maunabo, Patillas, and Las Piedras, Puerto Rico.

Characteristic	Category	*n*	%
Municipality			
	Maunabo	20	27
	Patillas	40	53
	Las Piedras	15	20
Age			
	<55	9	16
	55–64	17	30
	≥65	31	55
Monthly Income (USD)			
	Less than $500	9	16
	$501–$1000	18	31
	$1001–$1500	16	28
	$1501 or more	15	26
Sex			
	Man	75	100
Marital status			
	Single/Never Married	9	16
	Married or partnered	30	52
	Divorced/Separated	13	22
	Widower	6	10
Highest level of education			
	Incomplete High School	13	23
	Complete High School	26	46
	Technical degree	7	12
	Associate’s degree	1	2
	Bachelor’s degree	8	14
	Graduate‘s degree (Masters or Doctorate)	2	3

Note: The sociodemographic information of the Maunabo participants who attended the workshop was not collected.

## Data Availability

The data supporting the findings of this study are not publicly available due to ethical restrictions and confidentiality agreements submitted to the Institutional Review Board. This agreement was included in the informed consent forms signed by the study participants.
